# Rho GTPase Regulators and Effectors in Autism Spectrum Disorders: Animal Models and Insights for Therapeutics

**DOI:** 10.3390/cells9040835

**Published:** 2020-03-31

**Authors:** Daji Guo, Xiaoman Yang, Lei Shi

**Affiliations:** JNU-HKUST Joint Laboratory for Neuroscience and Innovative Drug Research, College of Pharmacy, Jinan University, Guangzhou 510632, China; daji0321@stu2017.jnu.edu.cn (D.G.); yxm3644@stu2018.jnu.edu.cn (X.Y.)

**Keywords:** Rho GTPase, autism spectrum disorder, guanine nuclear exchange factor, GTPase-activating protein, animal model, behavior

## Abstract

The Rho family GTPases are small G proteins that act as molecular switches shuttling between active and inactive forms. Rho GTPases are regulated by two classes of regulatory proteins, guanine nucleotide exchange factors (GEFs) and GTPase-activating proteins (GAPs). Rho GTPases transduce the upstream signals to downstream effectors, thus regulating diverse cellular processes, such as growth, migration, adhesion, and differentiation. In particular, Rho GTPases play essential roles in regulating neuronal morphology and function. Recent evidence suggests that dysfunction of Rho GTPase signaling contributes substantially to the pathogenesis of autism spectrum disorder (ASD). It has been found that 20 genes encoding Rho GTPase regulators and effectors are listed as ASD risk genes by Simons foundation autism research initiative (SFARI). This review summarizes the clinical evidence, protein structure, and protein expression pattern of these 20 genes. Moreover, ASD-related behavioral phenotypes in animal models of these genes are reviewed, and the therapeutic approaches that show successful treatment effects in these animal models are discussed.

## 1. Introduction

Autism spectrum disorder (ASD) is a neurodevelopmental disorder characterized by two core symptoms: (1) impaired social interaction and communication, and (2) repetitive or restricted interest and behaviors. The average global prevalence of ASD is ~0.62% [1], and studies in Europe and Asia have identified individuals with ASD with an average prevalence between 1% and 2% [2,3,4,5,6]. Statistics from CDC (Centers for Disease Control and Prevention)’s Autism and Developmental Disabilities Monitoring (ADDM) Network revealed that one in 59 children were diagnosed with ASD in United States in 2018 [7]. In addition, there is a high rate of co-occurring mental health disorders in ASD patients [8]. Meta-analysis of twin studies show that monozygotic twins have significantly higher concordance rate of ASD than dizygotic twins [9,10], thus the aetiology of ASD is closely related to genetic component. However, the genetic causes of ASD are very complex as a huge number of genes contribute to the pathogenesis of ASD. Therefore, databases of ASD-related genes, such as SFARI (Simons foundation autism research initiative) Gene [11,12] and AutDB (the autism gene database) [13], have been established. With the development of genome sequencing, increasing genes related to ASD have been identified. As of November 2019, more than 800 genes have been included in SFARI Gene [14] and more than 1000 genes have been listed in AutDB [15]. Among these ASD susceptibility genes, many converge on synapse regulation such as the regulation of development and maturation of synaptic contacts and synaptic transmission [16,17,18].

In the nervous system, precise neuronal connectivity depends on synapses. It is well known that dendritic spines, which are enriched with filamentous actin, are dynamic structures important for synapse formation, function and plasticity [19]. Rho family GTPases are key regulators of the actin cytoskeleton that play critical roles in axonal outgrowth, dendritic spine morphogenesis, and synapse formation [20]. The Rho family GTPases, which belong to the Ras superfamily, are small G proteins sized ~20 KDa. Human Rho family GTPases include 20 members that can be classified into eight groups [21,22]. By cycling between GTP-bound active forms and GDP-bound inactive forms, Rho GTPases regulate a diverse array of cellular events, including the control of growth, migration, adhesion, and differentiation. Rho GTPase activity is regulated by two different kinds of regulatory protein: guanine nucleotide exchange factors (GEFs), which catalyze the replacement of GDP by GTP, enabling the GTPases to recognize downstream effectors, and GTP-activating proteins (GAPs), which negatively regulate GTPase activity by favoring the GDP-bound forms [19,23]. Rho GTPase activity regulation is a complex process as 82 GEFs [24,25] and 66 GAPs (of which 57 have a common GAP domain) [26] have been identified so far in humans. The complexity of Rho GTPase signaling is also contributed by their downstream effectors, as there are over 70 downstream effectors identified to be capable of transducing signals from Rho GTPases [27].

**Figure 1:**
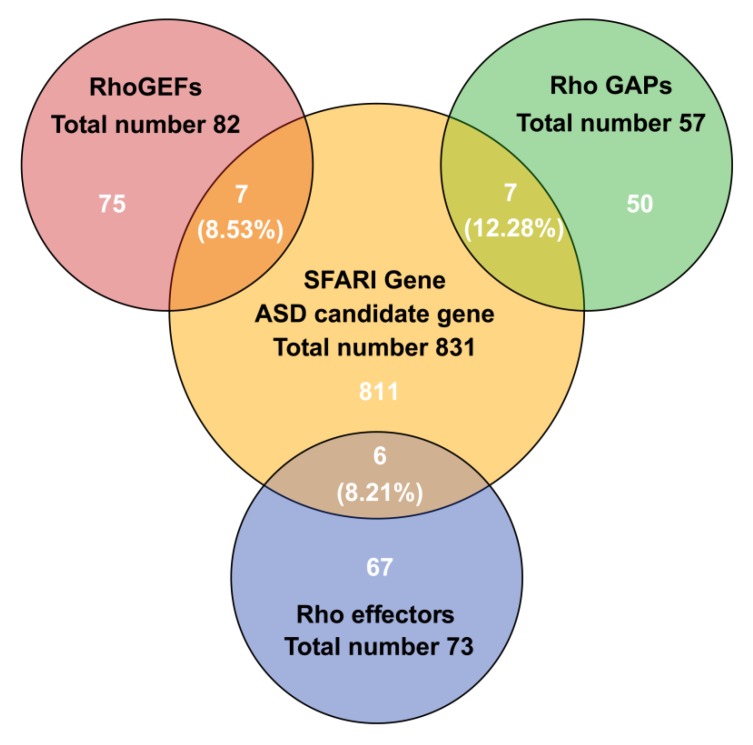
Overlap of human gene sets of RhoGEFs, RhoGAPs, and Rho effectors with autism spectrum disorder (ASD) risk genes in Simons foundation autism research initiative (SFARI).

A number of extensive review articles have summarized the impact of Rho family GTPases in neural development and diseases [23,28,29,30,31,32,33]. Moreover, multiple lines of evidence have suggested that Rho GTPase signaling may contribute to the pathogenesis of ASD. By searching SFARI Gene, it is notable to find that 8.53% (seven in 82) of RhoGEFs, 12.28% (seven in 57) of RhoGAPs and 8.21% (six in 73) Rho effectors are categorized as ASD-risk genes (Figure 1); 2.40% (20 in 831) ASD-risk genes directly participate in Rho GTPase signaling. We also find that all these genes encode regulatory proteins or effectors of three most-well studied Rho GTPases, Ras homolog family member A (RhoA), cell division cycle 42 (Cdc42), and Ras-related C3 Botulinum Toxin Substrate 1 (Rac1) (Table 1). In this review, the clinical evidence and animal models of these 20 genes are summarized. Moreover, therapeutic approaches that are capable of correcting the abnormalities caused by dysfunctions of these Rho GTPase regulators and effectors are discussed.

## 2. Rho Family GTPases and ASD

Rho GTPases themselves have been rarely reported as risk genes of ASD. The only evidence so far is the linkage of *RAC1* with ASD. Rac1 is an important Rho GTPase family member which regulates actin polymerization and spine remodeling through multiple signaling pathways, including PAKs (p21-activated kinases)-LIMK (LIM-domain-containing protein kinase)-cofilin [34], IRSp5 (insulin receptor substrate p53)-WAVE (Wiskott–Aldrich syndrome protein (WASP) family verprolin-homologous protein)-Arp2/3 [35,36], and PKA (protein kinase A) [37]. *RAC1*, encoding RAC1 and RAC1B, is a candidate gene for ASD listed in AutDB. Seven individuals with de novo mutations of *RAC1* were identified in patients of developmental disorders with divergent phenotypes [38]. One of these individuals displayed hyperactive behavior, two presented stereotypic movements, and one was diagnosed with autism [38]. Rac1 is highly expressed in embryonic cortex [39], and is ubiquitously expressed in the hippocampus, neocortex, thalamus, and cerebellum [40,41]. Rac1 is essential for the formation of three germ layers during gastrulation [42], and lack of which leads to embryonic lethality in *Rac1* knockout (KO) mice. To understand the brain function of Rac1, several *Rac1* conditional KO (cKO) mouse models have been constructed and studied. For instance, Foxg1-Cre mediated deletion of *Rac1* in the ventricular zone (VZ) of telencephalon [43,44,45], Nkx2.1-Cre-mediated deletion in medial ganglionic eminence (MGE) [46], and Nestin-Cre-mediated deletion in precursors of neurons and glia during early embryonic stage [35] were used to study the important roles of Rac1 for brain development. Moreover, three studies investigated the behavioral changes in *Rac1* cKO mouse models. Haditsch and colleagues generated a mouse model in which *Rac1* is deleted in pyramidal neurons by Cre under CamKIIα promoter to study the role of Rac1 in memory [47,48]. They demonstrated that loss of Rac1 in the hippocampus impairs long-term potentiation (LTP), and Rac1-deficient mice have impaired spatial memory and working or episodic-like memory [47]. They also found that the impaired working memory in these mice is due to prolonged memory retention or perseveration of the previously learned location [48]. Pennucci and colleagues generated a mouse model named Rac1N mice in which Rac1 is deleted in postmitotic neurons by Synapsin-I-Cre [49]. Rac1N mice show hyperactivity in several exploration tasks, impairment in spatial and working memory, and defects in retaining the context memory [49]. This study also reported failed synchronization of cortical networks in Rac1N mice by quantitative electroencephalogram (EEG). Moreover, spontaneous inhibitory synaptic currents (sIPSCs) are decreased in CA1 glutamatergic pyramidal cells in these mice [49]. However, these findings only focus on memory-related behaviors, but not the typical ASD-related ones such as social behaviors.

## 3. RhoGEF Family and ASD

There are 82 members of the human RhoGEF family, which are divided into two different subtypes: the classical Dbl family and the atypical Dock family [24]. So far, there are 71 members identified in Dbl RhoGEF family, which is characterized by a Dbl Homology (DH) domain, the catalytic GEF domain, and a Pleckstrin-Homology (PH) domain. The DH domain specifically catalyzes the exchange of GDP for GTP, whereas the role of PH domain varies considerably between different members, but is believed to facilitate the activation and localization of all Rho GTPases [25,50,51]. The Dock family, which contains 11 members, shows completely different structural features from the Dbl family. Dock family proteins have two main domains, the Dock homology region (DHR) 1 domain, which is responsible for phospholipid binding, and the DHR2 domain, which possesses the GEF activity [52]. Dock proteins are closely related to neurological disease [28,53]. By examining the overlap of RhoGEF genes and SFARI Gene, we find the following seven *RHOGEFs* as ASD-risk genes: *ARHGEF9*, *TRIO*, *DOCK8*, *PREX1*, *ARHGEF10*, *DOCK1*, and *DOCK4* (see Appendix A) (Table 1).

### 3.1. ARHGEF9 (SFARI Gene Score: 1, High Confidence)

**Figure 2:**
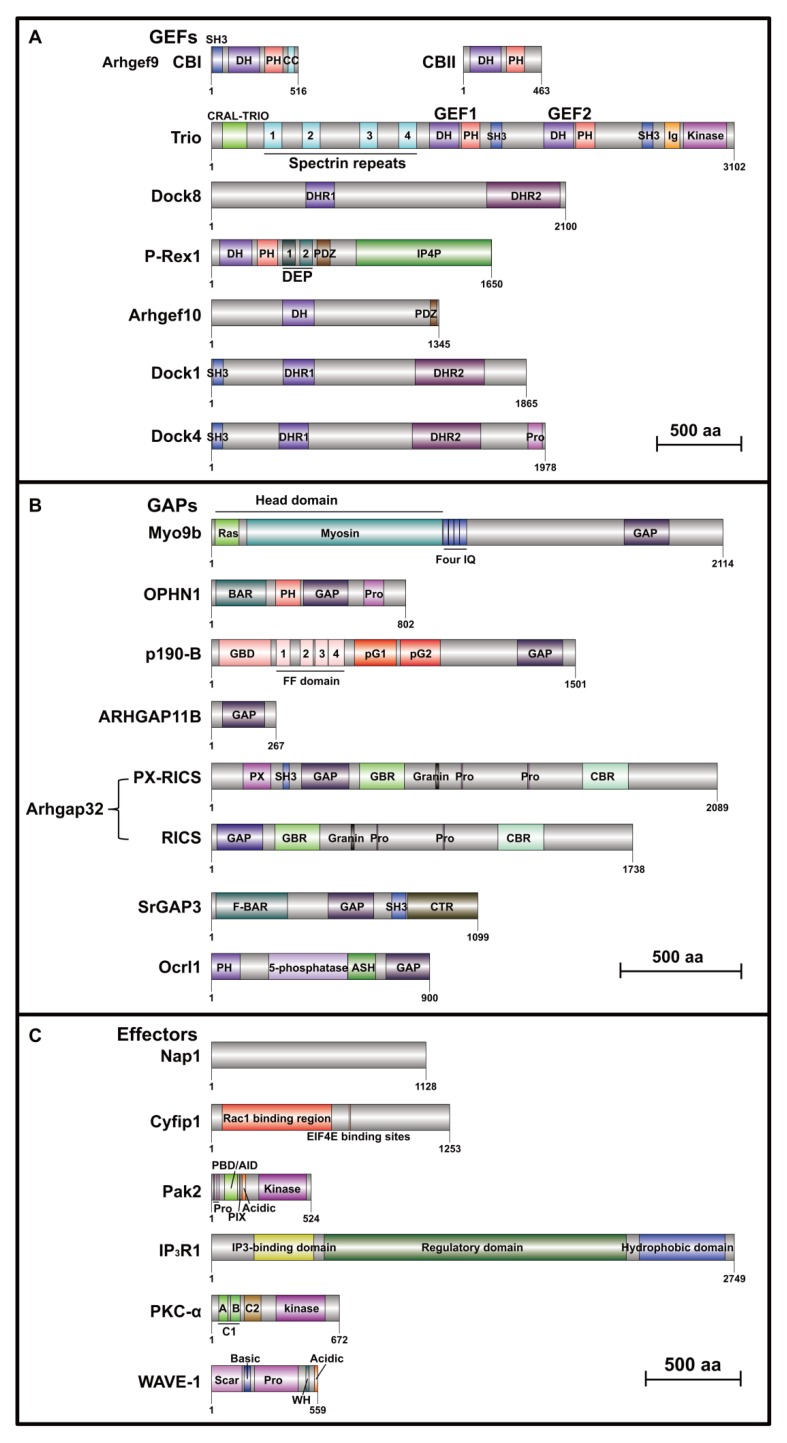
Schematics of protein domain structures of 20 ASD-related RhoGEFs, RhoGAPs, and Rho effectors. (**A**) Seven ASD-related RhoGEF protein domain architectures. Arhgef9 (which has two variants, CB I and CB II), Trio, P-Rex1, and Arhgef10 belong to Dbl family, which is characterized by a DH domain (dark violet) and a PH domain (light pink). Dock8, Dock1, and Dock4 belong to Dock family, which contains two main domains, DHR1 domain (dark orchid) and DHR2 domain (dark magenta). (**B**) Seven ASD-related RhoGAP protein domain architectures. In addition to a common catalytic GAP domain (purple), most RhoGAPs have multiple other functional domains. (**C**) Six ASD-related Rho effector protein domain architectures. All protein structures are generated using DOG 2.0 (Domain Graph, version 2.0) [94] based on corresponding mouse protein sequences except ARHGAP11B, for which human protein structure is shown as no homologs exist in rodents. Scales represent amino acid numbers of 500. AID, Autoinhibitory Domain; ASH, ASPM/SPD2/Hydin; BAR, Bin/Amphiphysin/Rvs; C1, binding site of diacylglycerol (DAG); C2, binding site of Ca^2+^; CBR, β-catenin-binding region; CC, coiled-coil; CRAL-TRIO, cellular retinaldehyde-binding protein and TRIO guanine exchange factor; CTR, C-terminal region with proline-rich; DEP, Disheveled, EGL-10, Pleckstrin; DH, Dbl Homology; DHR1, Dock homology region 1; DHR2, Dock homology region 2; F-BAR, Fes-Cip4 homology Bin/Amphiphysin/Rvs; FF, domain with two conserved phenylalanine residues; GAP, GTPase-activating proteins; GBD, guanosine triphosphate (GTP)-binding; GBR, GABARAP-binding region; Ig, immunoglobulin; IP4P, inositol polyphosphate 4-phosphatase; IQ, short calmodulin-binding motif containing conserved isoleucine and glutamine residues; PBD, p21-binding domain; PDZ, PSD95/SAP90, DlgA, ZO-1; pG1, pseudoGTPase domain 1; pG2, pseudoGTPase domain 2; PH, Pleckstrin-Homology; PIX, Pak-interacting exchange factor; Pro, proline-rich; PX, phox homology; Scar, Scar homology; SH3, Src homology 3; WH, WASF homology.

Rho guanine nucleotide exchange factor 9 (Arhgef9), also known as collybistin (CB), is a Dbl family GEF for Cdc42. *ARHGEF9* is located on chromosome Xq11.1-q11.2. The first report on the linkage of *ARHGEF9* with ASD identified a de novo microdeletion of Xq11.1 including entire *ARHGEF9* in a male patient, who presented with severe intellectual disability (ID), epilepsy, and mild to moderate autism [54]. A second de novo mutation of *ARHGEF9* was identified in a female patient diagnosed with ASD, ID and speech delay [55]. Subsequently, more de novo deletions of *ARHGEF9* were found in patients with ASD co-occurring with developmental delay (DD) or other mental disorders [56,57,58], suggesting that *ARHGEF9* is a strong candidate for ASD. Alternative splicing of *Arhgef9* transcripts creates two CB variants, I and II [59]. CB I has typical domains of Dbl family that include an Src homology 3 domain (SH3), a PH and a DH domain followed by a predicted coiled-coil (CC) domain, whereas CB II lacks SH3 and CC domains [59,60] (Figure 2A). CB is expressed predominantly in the brain, with enrichment in the gray matter, cerebral cortex, hippocampus, and cerebellum [59,61,62]. It was found that the CB1 level is high during early brain developmental stage, whereas CB2 expression maintains high and constant levels throughout brain development [60]. CB KO mice exhibited elevated anxiety levels and impaired spatial memory (Table 2; Supplementary Table S1), and showed reduced GABAergic transmission, increased LTP, and decreased long-term depression (LTD) in hippocampal CA1 region [63]. Two studies using this mouse line investigated electrophysiological characteristics in hippocampal dentate gyrus (DG) region, demonstrating that CB plays an important role in maintaining normal granule cell excitability, GABAergic network inhibition, and synaptic plasticity [64,65].

### 3.2. TRIO (SFARI Gene Score: 1, High Confidence)

Trio Rho guanine nucleotide exchange factor (Trio), a large protein of the mammalian Dbl family, activates both Rac1 and RhoA. *TRIO*, located on chromosome 5p15.2, is a strong candidate gene for ASD. By using whole-exome sequencing (WES) and transmission and de novo association (TADA) analysis of rare coding variations, a study identified *TRIO* as a gene strongly enriched for variants likely to affect autism risk from 3871 autism cases [95]. Subsequently, several whole-exome and genome sequencing studies identified several *TRIO* variations in subjects with autism [96,97] or ID co-occurring with autism [98,99]. Moreover, a large number of *TRIO* mutations leading to either reduced or excessive TIRO activity were found in ASD and neurodevelopmental disorder (NDD)/ASD/ID patients [100,101]. Trio protein contains two SH3 domains, a CRAL-TRIO (cellular retinaldehyde-binding protein and TRIO guanine exchange factor) domain, an immunoglobulin (Ig) domain, several spectrin-like repeats, two RhoGEF domains and a serine kinase domain [102] (Figure 2A). The GEF domain 1 activates Rac1 and RhoG, whereas the GEF domain 2 acts as an exchange factor for RhoA [103]. Because of the alternative splicing in *Trio*, Trio family is consisted of several isoforms, namely TrioA-E and Tgat [102,104,105]. Trio is enriched in the nervous system, and different isoforms are highly expressed in the cortex, hippocampus, striatum, and cerebellum, except for TrioC, which is highly expressed in the cerebellum [105]. Trio has important roles for embryonic development, as *Trio*^-/-^ mice die between embryonic day (E) 15.5 and birth [106]. Trio is also essential for brain development, because *Trio*^NKO^ mice, in which the *Trio* is deleted in neuronal and glia progenitors by Nestin-Cre, have high death rate after birth; hypoplasia was found in the residual survival individuals with disruptive development of the cerebellum [107]. Two recent studies on deletion of *Trio* in the forebrain showed that these cKO mice can survive to adulthood. In one study, *Emx1-Trio*^-/-^ mice in which *Trio* deletion is restricted to the cerebral cortex and hippocampus show deficient spatial learning [66] (Table 2; Supplementary Table S1). In another study, *Trio* was deleted by NEX-Cre in neocortex and hippocampus starting from E11.5 [108]. Both *NEX-Trio*^+/-^ and *NEX-Trio*^-/-^ mice displayed impaired social preference and impaired motor coordination, and anxious behaviors were observed in all *Trio* mutant mice except female *NEX-Trio*^-/-^ mice [67] (Table 2; Supplementary Table S1). Moreover, partial or full loss of Trio expression in motor cortex Layer 5 pyramidal neurons lead to disruption in presynaptic release probability, postsynaptic currents, and LTP [67]. However, the change in the ratio of NMDA ((N-methyl-D-aspartate)-type glutamate) receptor/AMPA (α-amino-3-hydroxy-5-methyl-4-isoxazolepropionic acid) receptor-mediated excitatory postsynaptic currents (EPSCs) was opposite in these two mouse lines [67]. These findings show comprehensive phenotypic changes on behaviors and neural function by *Trio* deficiency. It was also found in vitro that gain-of-function forms of *TRIO* variants lead to increased Rac1 activity and synaptic AMPA receptor function, whereas loss-of-function forms lead to decreased Rac1 activity and AMPA receptor function [100,101]. However, gain-of-function *Trio* mouse models have not been generated and investigated for ASD pathology thus far.

Katrancha and colleagues found decreased levels of PDE4A5, a negative regulator of PKA signaling, and corresponding increased PKA signaling in *NEX-Trio*^+/-^ and *NEX-Trio*^-/-^ motor cortex [67]. To examine approaches for correcting the abnormalities caused by *Trio* deficiency, they transfected *Trio*^+/flox^ neurons with GFP-P2A-Cre to establish *Trio* haploinsufficiency neurons as an in vitro model. These neurons showed increased spine density and decreased dendritic branch number, mimicking the phenotypes observed in *NEX-Trio*^+/-^ mouse brain [67]. Moreover, they treated the *Trio* deficient neurons with Rp-cAMPS, a competitive PKA antagonist, which reversed the increased dendritic spine density [67] (Table 3). For a non-pharmacological approach, PDE4A5 overexpression was capable of correcting the increased spine density but not branching deficits in *Trio*^+/-^ neurons [67] (Table 3). However, whether these two therapeutic approaches could rescue abnormal behaviors in *NEX-Trio*^+/-^ mice has not been investigated.

### 3.3. DOCK8 (SFARI Gene Score: 2, Strong Candidate)

Dedicator of cytokinesis 8 (Dock8) belongs to Dock-C subfamily, which lacks recognizable domains besides the DHR1-DHR2 module [28,110] (Figure 2A). Dock8 displays Cdc42-specific GEF activity [111,112]. *DOCK8* is located on chromosome 9p24.3, which is identified as a linkage region in large autism extended pedigrees [113,114]. Moreover, multiple variants of *DOCK8* are found in several genome sequencing studies on ASD patients [95,115,116,117,118,119]. Dock8 is primarily expressed in hematopoietic tissues, and Dock8 deficiency causes a combined immunodeficiency syndrome [110,112,120]. Recently, a study examined the expression of Dock8 in various cell types in the central nervous system (CNS), which reported that Dock8 is specifically expressed in microglia, but not neurons, astroglia, and retinal Müller glia [121]. A *Dock8* KO mouse line has been generated, which showed abnormal microglial activity in retina [121]. However, the role of Dock8 in regulating neural behaviors has not been explored yet.

### 3.4. PREX1 (SFARI Gene Score: 2, Strong Candidate)

Phosphatidylinositol-3,4,5-trisphosphate-dependent Rac exchange factor 1 (P-Rex1) is a member of Dbl family. *PREX1* is located on chromosome 20q13.13. A study on the Chinese Han population revealed that common variations in *PREX1* are found in autistic individuals, and *PREX1* mRNA levels are lower in the peripheral blood cells of autism subjects [68]. The main domain structures of P-Rex1 include a DH domain which has GEF activity, a PH domain which binds to PIP3 (phosphatidyl inositol (3,4,5) trisphosphate), two DEP (Disheveled, EGL-10, Pleckstrin) and two PDZ (PSD95/SAP90, DlgA, ZO-1) protein interaction domains, and a C-terminal domain which is similar to IP4P (inositol polyphosphate 4-phosphatase) [122] (Figure 2A). P-Rex1 activates several RhoGEF family members in vitro, but it only activates the Rac family and RhoG in vivo [123]. A study showed that P-Rex1 is expressed mainly in peripheral blood leukocytes and the brain in human [124]. In the developing mouse brain, the expression of P-Rex1 is present in the cerebral cortex, hippocampus, olfactory bulbs, and the cerebellum [125], and P-Rex1 is expressed in different types of cell, including neurons, neural precursor cells, and glial cells [125]. Thus, P-Rex1 may have multiple roles in the nervous system. A study reported several motor behavioral deficits in *Prex1*^-/-^, *Prex2*^-/-^ double-knockout mice [126]. A more recent study using a *Prex1* KO mouse line investigated the behavioral, electrophysiological and biochemical changes after P-Rex1 is absent. These *Prex1*^-/-^ mice display core ASD-like features, including impaired social novelty and social memory, decreased ultrasonic calls in pups, increased time in grooming, and disrupted behavioral inflexibility [68] (Table 2; Supplementary Table S1). Moreover, mice with specific knockdown P-Rex1 in hippocampal CA1 region recapitulate the social defects and disrupted behavioral inflexibility in *Prex1*^-/-^ mice [68]. The *Prex1*^-/-^ mice exhibit impairment in NMDA receptors-dependent LTD in hippocampal neurons [68].

To examine therapeutic approaches to rescue abnormal social behaviors, D-serine, a selective full agonist of the glycine modulatory sites on the NMDA receptors, was examined [68]. *Prex1*^-/-^ mice treated with D-serine restore normal behavior of social novelty in the Three-chamber test. Moreover, NMDA receptor-dependent LTD impairment in *Prex1*^-/-^ mice is restored after D-serine treatment [68] (Table 3). As *Prex1*^-/-^ mice show reduced Rac1 activity in the hippocampus [68], overexpression of Rac1 in the hippocampus of *Prex1*^-/-^ mice is able to correct the failure of social preference and reversal learning, and the disruption of NMDA receptors function [68] (Table 3). Furthermore, re-expressing P-Rex1 in *Prex1*^-/-^ *mice* also ameliorates impaired NMDA receptor-dependent LTD, deficient social behavior, and disruptive reversal learning [68] (Table 3).

### 3.5. ARHGEF10 (SFARI Gene Score: 3, Suggestive Evidence)

Rho guanine nucleotide exchange factor 10 (Arhgef10) belongs to the Dbl family, with a DH domain functioning as the GEF for RhoA and a putative PDZ-binding motif [127,128,129] (Figure 2A). *ARHGEF10* is located on chromosome 8p23.3. Several de novo and inherited missense variants in *ARHGEF10* have been identified in ASD patients [95,119,130]. Arhgef10 is ubiquitously expressed in the central and peripheral nervous system during embryonic development [131] and is widely expressed in the frontal cortex, striatum, hippocampus, and amygdala in adulthood [69]. An *Arhgef10* KO mouse line has been generated, and the mice display defective sociability and social novelty, hyperactivity, and reduced levels of anxiety and depression [69] (Table 2; Supplementary Table S1).

### 3.6. DOCK1 (SFARI Gene Score: 3, Suggestive Evidence)

Dedicator of cytokinesis 1 (Dock1), a member of the Dock-A family, activates Rac1 by its DHR2 domain [132]. *DOCK1* is located on chromosome 10q26.2. A genome-wide association study reported a loss-of-function variant of *DOCK1* in the affected proband as well as the ASD-affected mother, but not in the unaffected sibling [133]. A recent study reported that two autistic siblings have unbalanced translocation on chromosome 10 which leads to *DOCK1* deletion [134]. Dock1 consists of an SH3 domain, a DHR1 domain, and a DHR2 domain [132] (Figure 2A). The levels of Dock1 protein are downregulated during developmental stages in hippocampal neurons [135]. Dock1 has a critical role for embryonic development, and it has been shown that whole-body *Dock1* KO mice are perinatal or neonatal lethal [136]. There have been no *Dock1* cKO mouse models so far for the investigation of neuronal function and behavioral changes related to ASD.

### 3.7. DOCK4 (SFARI Gene Score: 3, Suggestive Evidence)

Dedicator of cytokinesis 4 (Dock4), a member of the Dock-B family, is an atypical Rac1 GEF. *DOCK4* is located on chromosome 7q31.1 which belongs to AUTS1 (designated as autism susceptibility locus 1), in which several ASD-associated genes reside. A comprehensive single nucleotide polymorphism (SNP) genotyping, association and copy number variation study in Caucasian autism families identified the linkage between *DOCK4* and ASD [137]. Several subsequent studies using SNP analysis reported multiple SNPs and chromosome microdeletions or duplications of *DOCK4* in autism and/or dyslexia patients [138,139]. A summary of *DOCK4* variations associated with ASD was provided in our previous study [70]. Dock4 contains an SH3 domain followed by a DHR1-DHR2 module, of which DHR2 is responsible for its GEF activity, and a proline-rich region [28] (Figure 2A). Dock4 is expressed at the highest level in the hippocampus, cortex, and cerebellum in adult rat brain [140], and the expression of Dock4 is upregulated along development in hippocampus in vivo and in hippocampal neurons cultured in vitro [140]. Our recent study used a *Dock4* whole-body KO mouse line to investigate the phenotypes in behaviors, synapse transmission, and molecular alterations. We found that *Dock4* KO mice display impaired social novelty preference, increased vocalizations, elevated anxiety levels, and disrupted spatial and working memory [70] (Table 2; Supplementary Table S1). Heterozygous (*Dock4* HET) mice also show defective social novelty preference and disrupted spatial memory in Y-maze [70] (Table 2; Supplementary Table S1). Both male and female mice were studied in this study, and the *Dock4* deficient mice show sex-dependent differences in anxiety levels and learning and memory. Notably, a small population of female *Dock4* KO and HET mice exhibit repetitive circling behaviors in home cage and open field arena [70] (Table 2; Supplementary Table S1). Moreover, mice with specific KO of *Dock4* in hippocampal CA1 region also exhibit defective social preference [70]. The hippocampal CA1 neurons of *Dock4* KO mice show impaired excitatory synaptic transmission especially NMDA receptor-dependent transmission, and decreased LTP [70].

As NMDA receptor impairment appeared to be responsible for the synaptic dysfunction in *Dock4* KO hippocampus, a widely used NMDA receptor agonist D-cycloserine (DCS) was used as a pharmacological therapeutic strategy. Indeed, social novelty in the Three-chamber test was restored in *Dock4* KO mice treated with DCS [70] (Table 3). For non-pharmacological therapeutic approaches, overexpressing Rac1 in hippocampus of *Dock4* KO mouse corrects defective social preference and disruptive NMDA receptor function [70] (Table 3). Moreover, overexpressing Rac1 in cultured Dock4-knockdown hippocampal neurons also reverses the decreased spine density [109] (Table 3).

## 4. RhoGAP Family and ASD

To date, 66 RhoGAPs have been identified, most of which contain a common RhoGAP domain that has the catalytic GAP activity [141]. In addition, almost all RhoGAPs have at least two to three additional domains, which may interact with different proteins and are thus engaging the RhoGAPs in different signaling pathways [30,141]. RhoGAPs play irreplaceable roles in axonal and dendritic development, and synaptic plasticity [19,30,31], disruption of which may contribute to the pathological mechanism of ASD. By overlapping 57 GAP domain-containing RhoGAP genes [26] with SFARI Gene, we find eight *RHOGAPs* as ASD-risk genes: *MYO9B*, *OPHN1*, *ARHGAP5*, *ARHGAP11B*, *ARHGAP32*, *SRGAP3*, and *OCRL* (Table 1).

### 4.1. MYO9B (SFARI Gene Score: 2, Strong Candidate)

Myosin IXB (Myo9b), a unique member of myosin family, contains a RhoGAP domain in its C-terminal tail, which stimulates the GTP hydrolysis of RhoA but not Cdc42 or Rac1 in vitro [142,143]. *MYO9B* is located on chromosome 19p13.11. Using WES and TADA analysis of rare coding variations of autism patients, *MYO9B* was identified as a gene strongly enriched for variants likely to affect autism risk [95]. Myo9b has a three-part structure: a head domain, four calmodulin-binding motifs containing conserved isoleucine and glutamine residues (IQ motifs) in the neck, and a RhoGAP tail [142,144] (Figure 2B). Human MYO9B is highly expressed in the immune system, and has minor levels in the respiratory system, digestive system, reproductive system, and nervous system [145]. As *MYO9B* is related to inflammatory bowel diseases [146] and celiac disease [147], the use of *Myo9b* KO mice has been mostly limited in studies of immune cells [148,149]. The role of Myo9b in the nervous system has been so far investigated in one study, which reported that Myo9b expression in the cerebral cortex reaches peak at around E18, and is decreased during development [150]. Knockdown of Myo9b in cultured cortical neurons or in developing cortex results in decreased dendrite length and number [150]. Nonetheless, the function of Moy9b in regulating neural behaviors has not been explored.

### 4.2. OPHN1 (SFARI Gene Score: 2, Strong Candidate)

Oligophrenin 1 (OPHN1) is a RhoGAP family member that is capable of inhibiting RhoA, Rac1, and Cdc42 in vitro without any specificity [151]. *OPHN1* is located on chromosome Xq12 and is closely related to mental retardation (MR) and cerebellar hypoplasia [152]. Rare missense variants and rare hemizygous deletions in *OPHN1* have been identified in ASD patients in different studies [153,154]. In recent exome sequencing studies, several de novo and maternally inherited variants of *OPHN1* have been found in ASD patients with other mental disorders [99,155]. OPHN1 possesses a Bin/Amphiphysin/Rvs (BAR) dimerization domain and a PH domain at the N-terminus, followed by the GAP domain and the proline-rich region at the C-terminus [156,157] (Figure 2B). OPHN1 is ubiquitously expressed with highest levels in various brain regions, including the olfactory bulb, frontal lobes, sensory cortex, hippocampus, thalamus, and cerebellum [151,157], and its brain expression remains high throughout development [150,151,157]. Due to the central role of OPHN1 for maintaining dendritic spines, *Ophn1* KO mice (*Ophn1*^-/y^) have been generated for studying its in vivo function. *Ophn1*^-/y^ mice show hyperactivity, decreased behavioral lateralization in paw preference test, altered spatial memory [71], and impaired object recognition memory [72] (Table 2; Supplementary Table S1). Furthermore, a study showed that *Ophn1*^-/y^ mice are impaired in olfactory behavior [73], but another study reported that olfactory function is normal in the mice during social memory test [71] (Table 2; Supplementary Table S1). Lack of *Ophn1* leads to decreased paired-pulse facilitation (PPF) in hippocampal CA1 neurons [71] and increased miniature inhibitory postsynaptic current (mIPSC) amplitude and frequency in olfactory neurons [73]. A further study revealed that *Ophn1*^-/y^ mice exhibit disruptive presynaptic plasticity at cortico-lateral amygdala and hippocampal synapses in a PKA dependent manner [74]. The abnormal presynaptic function leads to deficient fear memory extinction in *Ophn1*^-/y^ mice [74] (Table 2; Supplementary Table S1). Another recent study demonstrated that *Ophn1*^-/y^ mice display cognitive impairment in Y-maze spatial working memory test with high occurrence of perseverative behaviors [75] (Table 2; Supplementary Table S1), which suggests poor behavioral flexibility in *Ophn1*^-/y^ mice. This study also found that *Ophn1*^-/y^ mice show decreased vicarious trial and error (VTE) behavior [75] (Table 2; Supplementary Table S1), which is a pause and look back and forth behavior reflecting a deliberation process during decision making in rodents [158].

Interestingly, the abnormal behaviors observed in *Ophn1*^-/y^ mice are found to be resulted from PKA-dependent dysfunction of medial prefrontal cortex (mPFC) neuronal networks [75]. Consistently, increasing PKA activity in mPFC of WT mice causes similar impairments in Y-maze as observed in *Ophn1*^-/y^ mice [75]. Hence, local infusion of Rp-cAMPS, a competitive PKA antagonist, into mPFC of *Ophn1*^-/y^ ameliorates the spatial working memory deficits [75] (Table 3). Moreover, RhoA and its effector Rho kinase (ROCK) are well-studied downstream mediators of OPHN1 in vivo. As over-activation of the RhoA-ROCK pathway was observed in *Ophn1*^-/y^ mice [73], fasudil, a clinically approved inhibitor of ROCK as well as PKA, has been examined. Chronic fasudil treatment in *Ophn1*^-/y^ mice is able to reverse the alterations of spine morphology and mIPSC in olfactory neurons, and restore olfactory behaviors [73] (Table 3). Furthermore, chronic fasudil treatment restores fear memory extinction, locomotor activity, and object recognition memory in *Ophn1*^-/y^ mice (Table 3), but it does not correct the abnormal working and spatial memory in these mice [72,74]. The abnormal brain morphology in *Ophn1*^-/y^ mice, including the enlargement of brain lateral ventricles and the increase in hippocampal mushroom-shaped spines, are all ameliorated by fasudil treatment [72] (Table 3).

### 4.3. ARHGAP5 (SFARI Gene Score: 3, Suggestive Evidence)

Rho GTPase activating protein 5 (Arhgap5), also known as p190-B, is a member of the RhoGAP family that acts on inhibiting RhoA, Cdc42 and Rac1 [159,160]. *ARHGAP5* is located on chromosome 14q12. Several de novo mutations in *ARHGAP5*, which lead to loss-of-function or missense variants, are found in genome sequencing studies [95,96,161]. From N- to C-terminus, p190-B is composed of a guanosine triphosphate (GTP)-binding domain (GBD), four FF domains, which characterize two conserved phenylalanine residues in each domain, two pseudoGTPase domains (pG1 and pG2), and a GAP domain [162,163] (Figure 2B). p190-B is highly expressed in the brain, stomach, and thymus [164]. A study on the expression pattern of RhoA GAPs shows that the protein level of p190-B is increased in the cerebral cortex during postnatal development [150]. p190-B plays an important role during embryonic development in the CNS [165,166] and hematopoietic system [167,168], and homozygous deletion of p190-B results in perinatal lethality [164]. However, mouse models in which p190-B is deficient in CNS are still missing for investigation of this protein on regulating neural function and behaviors.

### 4.4. ARHGAP11B (SFARI Gene Score: 3, Suggestive Evidence)

Rho GTPase activating protein 11B (ARHGAP11B) is a truncated version of ARHGAP11A, which contains 267 amino acids (aa) mostly comprised of a truncated GAP-domain and a unique C-terminal sequence [169] (Figure 2B). *ARHGAP11B* is located on chromosome 15q13.2. Copy number variations of *ARHGAP11B* are found in patients with autism, ID [170], and schizophrenia (SCZ) [171]. Besides, a rare deletion overlapping *ARHGAP11B* is identified in monozygotic twins with SCZ [172]. Another study on 1257 autistic patients reported that loss of *ARHGAP11B* is detected in eight patients, and two of them carry de novo deletions of *SHANK2*, a high risk gene of ASD [173]. *ARHGAP11B* is a human-specific gene with an important role in human neocortex expansion [174,175], especially for the amplification of basal radial glial cells. However, ARHGAP11B does not exhibit RhoGAP activity in vivo [174,176], thus it may not truly belong to RhoGAP family. As there is no homolog gene of *ARHGAP11B* in rodents, it is not possible to investigate its function using rodent models.

### 4.5. ARHGAP32 (SFARI Gene Score: 3, Suggestive Evidence)

Rho GTPase activating protein 32 (Arhgap32), also designated as RICS, Grit, p200RhoGAP, p250RhoGAP, or GC-GAP, is a member of RhoGAP family. *ARHGAP32* is located on chromosome 11q24.3. In a study of 17 patients with Jacobsen syndrome, also called 11q terminal deletion disorder, eight patients with autistic behaviors have 8.7–14.6 Mb deletions of chromosome 11q, affecting four genes including *ARHGAP32* [177]. Another study on 1543 Chinese ASD probands discovered the first de novo LGD (likely gene-disrupting) mutation in *ARHGAP32* [115]. Recently, a study using single-molecule molecular inversion probes on ASD patients found an inherited mutation of *ARHGAP32* [118]. Some studies showed that RICS possesses GAP activity toward Cdc42, Rac1, and RhoA equally [178,179], while others reported that RICS prefers RhoA and Cdc42 [180,181] or even only RhoA [182] as its substrate in vitro. RICS contains five domains, a GAP domain, a GABARAP-binding region (GBR), a granin motif (Granin), a polyproline stretch (Pro-rich), and a β-catenin-binding region (CBR) [183] (Figure 2B). RICS is abundant in the nervous system [178,179,180,181,182], especially in the cerebral11111 cortex, amygdala, thalamus, and hippocampus [181,184]. The expression of RICS in mouse brain reaches to peak at about postnatal day (P) 12 during development, and is downregulated afterwards [185]. Similar expression pattern of RICS is also observed in cultured hippocampal neurons [185]. Immunofluorescent staining of cultured hippocampal neurons and immunoblotting of subcellular fractionations reveal that RICS is concentrated in the postsynaptic density [179,181]. Another longer spliced isoform of RICS, namely PX-RICS, has been reported, which has an additional phox homology (PX) domain and an SH3 domain in its N-terminal region [186] (Figure 2B). PX-RICS protein is also predominantly expressed in the nervous system [186], and at relatively low levels in the lung, kidney and spleen [186]. A *RICS* KO (*PX-RICS*^-/-^) mouse line has been generated, in which both RICS and PX-RICS protein are absent [184]. *PX-RICS*^-/-^ mice exhibit defective social novelty preference, reduced passive social interaction, decreased ultrasonic calls in pups, increased repetitive behaviors, poor behavioral flexibility, impaired motor coordination, a seizure-prone phenotype, and abnormal cued fear learning memory [76,77] (Table 2; Supplementary Table S1). Moreover, *PX-RICS*^+/−^ mice also exhibit moderate defects in social and repetitive behavior [76] (Table 2; Supplementary Table S1). Notably, PX-RICS-deficient hippocampal CA1 neurons show decreased mIPSC amplitude, suggesting impaired GABA_A_R-mediated synaptic transmission in *PX-RICS*^-/-^ mice [76]. Hence, clonazepam (CZP), a benzodiazepine agonist of GABA_A_R, was examined as a treatment for *PX-RICS*^-/-^ mice. Indeed, CZP administration leads to restoration of normal social preference and improved reversal learning and cued fear learning memory [76,77] (Table 3).

### 4.6. SRGAP3 (SFARI Gene Score: 3, Suggestive Evidence)

SLIT-ROBO Rho GTPase activating protein 3 (SrGAP3), also called mental disorder-associated GAP protein (MEGAP) and WAVE-associated RacGAP protein (WRP), is a member of RhoGAP family for Rac1 and Cdc42 but not RhoA [187,188]. *SRGAP3* is located on chromosome 3p25.3. Two de novo missense variants in *SRGAP3* are identified in ASD probands from a genome sequencing study [161]. *SRGAP3* is also listed as a neurodevelopmental-disorder risk gene in ID patients co-occurring with autism [98]. SrGAP3 has four main domains including an N-terminal Fes-Cip4 homology Bin/Amphiphysin/Rvs (F-BAR) domain, a central GAP domain for its Rac1-GAP activity, a C-terminal SH3 domain, and a C-terminal region (CTR) with proline-rich motif [189,190] (Figure 2B). Both human and mouse SrGAP3 is widely expressed in the whole CNS during embryonic development [187,191,192], and highly expressed in the hippocampus, amygdala, thalamus, cortex, and cerebellum of the adult brain [187,193]. So far, two SrGAP3-deficient mouse models have been studied. First, a conditional *SrGAP3* KO mouse model named *WRP* KO mice was generated, in which *SrGAP3* is deleted by Nestin-Cre [78]. The *WRP* HET and KO mice both show impaired long-term memory and spatial memory in several behavior tests, and also display abnormal reversal learning in Morris water maze [78] (Table 2; Supplementary Table S1). Second, a SrGAP3-deficient mouse (*SrGAP3*^-/-^ mice) was generated, in which an N-terminal 141 aa of SrGAP3 protein instead of the full length protein was expressed, mimicking the *SRGAP3* deletion of a patient with severe ID [79]. The *SrGAP3*^-/-^ male mice display hypoactivity, abnormal social exploration, and impaired working memory, whereas female mice show normal locomotion but severely impaired social behaviors (Table 2; Supplementary Table S1) and decreased prepulse inhibition (PPI) [79]. However, another study using this mouse model reported normal locomotion but reduced marble burying in *SrGAP3*^-/-^ male mice [80] (Table 2; Supplementary Table S1).

### 4.7. OCRL (SFARI Gene Score: S, Syndromic)

Inositol polyphosphate 5-phosphatase OCRL (Ocrl1), which encodes a type II phosphatidylinositol bisphosphate (PtdIns4,5P_2_) 5-phosphatase, is a RhoGAP family member for Rac1 and Cdc42 [194]. *OCRL* is located on chromosome Xq26.1. Mutations in *OCRL* are related to Lowe syndrome, a multisystem disorders affecting eyes, the nervous system, and kidney [195]. An assessment of 52 male patients with Lowe syndrome using the Autism Screening Questionnaire found that 71.2% of patients met the cut-off score for ASD [196]. Moreover, a study has identified a full gene duplication of *OCRL* in a male ASD patient [197]. Ocrl1 has four main domains including a PH domain, a central 5-phosphatase domain, an ASPM/SPD2/Hydin (ASH) domain, and a C-terminal RhoGAP domain [198,199] (Figure 2B). Human OCRL1 is widely expressed in different tissues, with the highest levels observed in the brain, liver, and kidney [81]. A study showed that mice deficient with Ocrl1 alone fail to recapitulate the abnormalities observed in human [81] (Table 2; Supplementary Table S1). However, another mouse model with deletion of both *Ocrl1* and another type II PtdIns4,5P_2_ 5-phosphatase *Inpp5b*, but overexpression of human *INPP5B* display disorders related to Lowe syndrome [200]. Moreover, these mice show dysfunctional locomotor activity caused by muscular defects but normal sociability and learning memory [82] (Table 2; Supplementary Table S1), suggesting that this mouse model may not be used as an ASD model.

## 5. Rho GTPase Effectors and ASD

It is well known that Rho GTPases act as molecular switches that transduce upstream signals to downstream effectors to engage specific signaling cascades. Once in the GTP-bound active forms, the conformations of effector-binding regions of Rho GTPases are changed to allow interaction with the effectors [201]. This interaction regulates the function of effectors, resulting in a series of cell responses to the initial stimuli. There are a large number of molecules involved in Rho GTPase signaling, and more than 70 proteins have been identified as potential effectors of RhoA, Rac1, and Cdc42 [27]. We examine the overlap between these effector genes and SFARI Gene and find the following six effectors as ASD-risk genes: *NCKAP1*, *CYFIP1*, *PAK2*, *ITPR1*, *PRKCA*, and *WASF1* (Table 1).

### 5.1. NCKAP1 (SFARI Gene Score: 1, High Confidence)

Nck-associated protein 1, also known as Nap1, is a component of the WAVE1/2 complex that interacts with activated Rac1 [202]. *NCKAP1* is located on chromosome 2q32.1. A de novo LGD mutation of *NCKAP1* has been identified in ASD probands [203]. Subsequently, several de novo or maternally inherited mutations in *NCKAP1* are found in multiple WES studies on ASD probands [95,161,204]. Recently, two studies on Chinese and Caucasian ASD cohorts found more de novo LGD mutations in *NCKAP1*, which suggests that *NCKAP1* is a strong candidate gene for ASD [115,205]. Nap1 does not contain any known functional motif [206] (Figure 2C). Human Nap1 is extensively expressed in multiple tissues except peripheral blood leukocytes, with highest expression detected in the brain, heart, and skeletal muscle [207]. The expression of human Nap1 is ubiquitously observed in all brain regions, with relatively higher levels in the cerebellum, hippocampus, and amygdala [207]. A study reported that human Nap1 is preferentially expressed in neuronal cells and may participate in neuronal apoptotic pathway [207]. Immunoblots from different embryonic ages indicate a pattern of developmentally increased Nap1 expression in the mouse cerebral cortex [208]. Yokota and colleagues found that knockdown of endogenous Nap1 leads to defective neuronal differentiation in mouse cortical neurons [208]. They then generated a Nap1 mutant mouse line in which the N-terminal 898 aa of Nap1 fused with a 1291 aa β-geo reporter is expressed instead of the full-length protein [208]. However, these Nap1 mutant mice are embryonic lethal from E8.5–E10.5 due to neural tube and neuronal differentiation defects [208]. Therefore, mouse models of Nap1 deficiency are still absent for the investigation of Nap1 on regulating neural behaviors.

### 5.2. CYFIP1 (SFARI Gene Score: 2, Strong Candidate)

Cytoplasmic FMR1 interacting protein 1 (Cyfip1), also named Shyc and Sra1, is a Rac1-interacting protein and a partner of the WAVE complex that regulates actin filament. *CYFIP1* is located on chromosome 15q11.2. Several studies have reported that patients with 15q11.2 microdeletions and microduplications between breakpoints 1 and 2, which encompass several genes including *CYFIP1*, are diagnosed with neurodevelopmental disorders including ASD, ID, SCZ, attention-deficit/hyperactivity disorder (ADHD), and obsessive-compulsive disorder (OCD) [209,210,211,212]. A paternally inherited rare variant of *CYFIP1* is found in an autistic patient with a de novo *SHANK2* deletion [173]. Another paternally inherited rare variant of *CYFIP1* is found in a study on high functioning ASD patients [213]. Additionally, SNPs in *CYFIP1* were reported to correlate with ASD in two studies [214,215]. A study on *CYFIP1* mRNA expression in human dorsolateral prefrontal cortex revealed higher expression of *CYFIP1* mRNA in ASD and classical autism patients, and identified several common variants of *CYFIP1* in these patients [216]. A recent study also demonstrated significant increase in *CYFIP1* transcripts in the peripheral blood of ASD patients [217]. All these clinical findings reveal that altered *CYFIP1* dosage may contribute to the pathology of ASD. Cyfip1 has been shown to interact with Rac1, Fragile X mental retardation 1 (FMRP), and eukaryotic translation initiation factor 4E (EIF4E) [218,219] (Figure 2C). Cyfip1 is widely expressed in multiple tissues but not liver during development and is highly enriched in the hippocampus, cerebral cortex, cerebellum, olfactory bulb, and lateral septum in the adult brain [220]. Levels of Cyfip1 are high in the cortex and cerebellum at the stages of postnatal development, peaked at P23 and slightly decreased afterwards [221].

Four different strategies have been used for generation of *Cyfip1* KO mice. However, because Cyfip1 is very important for early embryonic development, none produce homozygous *Cyfip1* KO mice. Therefore, *Cyfip1* HET mice are used for studying behavioral and neural phenotypes in these studies. The first mouse model was generated by mutagenesis with a gene trap vector inserted into intron 1 of *Cyfip1*. There are no *Cyfip1* KO embryos in breeding [83]. *Cyfip1* HET mice display normal learning and memory abilities in several memory tests, but show more rapid extinction in inhibitory avoidance test, a test for hippocampus-dependent memory [83] (Table 2; Supplementary Table S1). These results indicate that the accuracy of memory processing in HET mice is much poorer. *Cyfip1* HET mice show increased mGluR-dependent LTD and abnormal presynaptic function in hippocampal slices [83,222]. The second one is generated by deleting exons 4–6 of *Cyfip1*. Inbreeded from *Cyfip1*^HET^ mice, fertilized *Cyfip1* KO oocytes are detectable in blastocyst stage [223]. The KO embryos can survive until E8.5, but become lethal due to complete developmental failure [223]. The *Cyfip1*^HET^ mice show absence of interest for social cues and deficits in motor learning [84] (Table 2; Supplementary Table S1). The third one is generated by deleting exon 5 of *Cyfip1*, and the *Cyfip1* homozygous KO embryos die before E9.5 [85]. Moreover, the difference between the maternal (m−/p+) and paternal (m+/p−) deficiency of *Cyfip1* was investigated. Interestingly, *Cyfip1* m−/p+ mice only display hypoactivity, whereas *Cyfip1* m+/p− mice display increased freezing in cued fear conditioning and abnormal transitions in zero-maze test [85] (Table 2; Supplementary Table S1). Both *Cyfip1* m-/p+ mice and *Cyfip1* m+/p- mice show reduced field EPSC and increased PPF in hippocampal CA1 region, and enhanced mGluR-dependent LTD is observed in *Cyfip1* m+/p- mice hippocampal CA1 neurons [85]. The fourth one is generated by inserting a gene trap cassette between exon 12 and 13 of *Cyfip1*. *Cyfip1*^+/-^ mice display impaired motor coordination, deficient sensory processing/novelty seeking behavior, reduced PPI, and decreased sensory motor gating [86] (Table 2; Supplementary Table S1), recapitulating some ASD and SCZ-like behavioral phenotypes. *Cyfip1*^+/-^ mice show reduced spontaneous neuronal activity and presynaptic function in cortical slices [86]. Besides the whole-body mutant mice, one *Cyfip1* cKO mouse model (*Cyfip1*^NEX^ cKO mice) has been generated, in which exon 4–6 of *Cyfip1* was deleted in forebrain excitatory neurons by NEX-Cre. These mice are viable until adulthood with no obvious abnormalities [224]. *Cyfip1*^NEX^ cKO mice show increased mIPSC amplitude in hippocampal CA1 neurons and display similar deficits in dendrite morphology and spine maturation to those described in *Cyfip1* haploinsufficient models [224]. There is also a *Cyfip1* mutant rat model generated by CRISPR/Cas9 technology, in which a 4 bp heterozygous deletion is introduced in exon 7 of *Cyfip1*, causing premature stop of the protein [87]. These *Cyfip1* haploinsufficient rats exhibit normal learning ability during all behavioral tests, but show deficits in behavioral flexibility [87] (Table 2; Supplementary Table S1). As increased transcript levels of *CYFIP1* is found in some ASD patients, two transgenic (Tg) *Cyfip1* mouse lines (Tg line 1 and Tg line 2) were generated by overexpressing human *CYFIP1*. mRNA of human *CYFIP1* was increased in the cortex and hippocampus of both Tg lines; Cyfip1 protein is also increased in the two brain regions of Tg line 1 and the hippocampus of Tg line 2, but not in the cortex of Tg line 2 [88]. Behaviorally, Tg line 2 mice display subtle defects of spatial learning memory and obvious increased fear response in contextual and cued fear conditioning test, whereas Tg line 1 mice show normal spatial learning memory and increased freezing only to the tone in the novel context during fear conditioning test [88] (Table 2; Supplementary Table S1). However, both Tg lines show no deficits in core ASD-related behaviors such as social and repetitive behaviors [88] (Table 2; Supplementary Table S1). Together, *Cyfip1* deficient mice or rats recapitulate core features of ASD, whereas *Cyfip1* Tg mice may not be appropriate ASD models.

As increased mGluR activation was caused by *Cyfip1* deficiency, mGluR1 inhibitor LY367385 and mGluR5 antagonist MPEP (2-Methyl-6-(phenylethynyl) pyridine) were used together. Indeed, these mGluR blockers normalized the mGluR-LTD to control levels in hippocampal slices of *Cyfip1* HET mice [83] (Table 3). However, the effect of these two inhibitors on behaviors has not been investigated. For non-pharmacological therapeutic approaches, motor training increased the number of newly formed dendritic spines in both WT and *Cyfip1*^HET^ mice, and the motor learning deficits in *Cyfip1*^HET^ mice can be alleviated by the behavioral training in early development but not in adult [84] (Table 3).

### 5.3. PAK2 (SFARI Gene Score: 2, Strong Candidate)

P21 activated kinase 2 (Pak2), activated by Rac1 and Cdc42, is a member of the group I PAK family that belongs to a family of serine/threonine kinases. *PAK2* is located on chromosome 3q29, which is classified as one of six strong autism risk loci [225]. A study on six patients of 3q29 microdeletion syndrome identified a ~1.5 Mb microdeletion, which includes entire *PAK2*. Two of these patients displayed autistic features [226]. Another study on two patients of 3q29 microdeletion syndrome with common ID, a history of autism, and other psychiatric symptoms also reported the same length deletion [227]. In a subsequent study, 11 of 44 patients with 3q29 microdeletion syndrome were found to display diverse neurodevelopmental disorders including autism [228]. Moreover, one de novo copy-number deletion containing *PAK2* was found in patients with ASD from the Simons Simplex Collection, and one de novo nonsense mutation and two inherited missense mutations were also found in *PAK2* in 914 Han Chinese patients with ASD [89]. All these four studies reveal that *PAK2* is a strong candidate for ASD. Pak2 has several recognized domains including two proline-rich regions and an AID (Autoinhibitory Domain) overlapping the PBD (p21-binding domain) in the N-terminal region, a kinase domain at the C-terminus, an acidic region, and a PIX (Pak-interacting exchange factor) binding site [229,230,231] (Figure 2C). Pak2 is ubiquitously expressed in multiple tissues [232]. Human PAK2 shows high expression levels in the brain during the fetal period and low levels after birth. Mouse Pak2 is also down-regulated at the postnatal development in the cortex [89]. Loss of *Pak2* leads to embryonic lethality at ~E8 [232,233,234]. Therefore, a recent study used *Pak2*^+/-^ mice to investigate the behavioral and neural functional changes caused by *Pak2* haploinsufficiency. This study revealed that *Pak2*^+/-^ mice display repetitive and stereotyped behaviors, impaired social interaction, social avoidance, reduced social preference index, and disruptive social memory [89] (Table 2; Supplementary Table S1). *Pak2*^+/-^ mice also exhibit decreased LTP in hippocampal CA1 region neurons [89]. Mechanistically, phosphorylation of both LIMK1, a major downstream target of group I PAKs, and its substrate cofilin were markedly decreased, suggesting abnormal LIMK1/cofilin-mediated actin polymerization in adult *Pak2*^+/-^ mice cortex [89]. To normalize endogenous p-cofilin levels in the cortex and hippocampus, a p-cofilin peptide was intravenously injected into adult *Pak2*^+/-^ mice as a substrate to compete with endogenous p-cofilin for phosphatases. As a result, this peptide moderately improved social behaviors but not repetitive behaviors in adult *Pak2*^+/-^ mice [89] (Table 3).

### 5.4. ITPR1 (SFARI Gene Score: 3, Suggestive Evidence)

Inositol 1,4,5-trisphosphate receptor type 1, also known as IP_3_R1, is a RhoA effector. IP_3_R1 is a member of IP_3_Rs, which are Ca^2+^ release channels on the endoplasmic reticulum. *ITPR1* is located on chromosome 3p26.1. Three de novo missense variants in *ITPR1* are found in ASD probands from WES studies [95,115,161]. In addition, a study on autism risk genes in probands from the Autism Clinical and Genetic Resources in China (ACGC) identified one maternally and one paternally inherited missense variant of *ITPR1* [118], and another variant is found in patients with NDDs [235]. IP_3_R1 has three main domains including a large N-terminal IP_3_-binding domain, a short C-terminal hydrophobic domain, and an intervening regulatory domain [236] (Figure 2C). The expression of IP_3_R1 is increased during embryogenesis [237]. Moreover, IP_3_R1 is predominately expressed in the nervous system [238,239] and is expressed in a wide range of brain regions including the cerebellum, cerebral cortex, hippocampus, olfactory bulb, globus pallidus, and striatum [238]. It was reported that homozygous *IP_3_R1*-deficient mice mostly die during the embryonic stage, and the born mice have severe ataxia and seizures and die at around P21 [240]. *IP_3_R1*^+/-^ mice also show deficits in motor coordination [90] (Table 2; Supplementary Table S1). To study the neural function of IP_3_R1, several *IP_3_R1* brain cKO mouse lines have been generated. A study reported that *L7-Cre;Itpr1*^flox/flox^ mice, in which Itpr1 is deleted in Purkinje cells, exhibit cerebellar ataxia at around 6 weeks and severe ataxia at 8 weeks after birth [91] (Table 2; Supplementary Table S1). *L7-Cre;Itpr1*^flox/flox^ mice could survive to adulthood, but display abnormal motor learning ability [91] (Table 2; Supplementary Table S1). In another study, three cKO lines were used, in which *Itpr1* deletion was restricted to the cerebral cortex and hippocampus (*Emx1-Cre;Itpr1*^flox/flox^ mice), the cerebellum and brainstem (*Wnt1-Cre;Itpr1*^flox/flox^ mice), and the caudate putamen and globus pallidus (*Gpr88-Cre;Itpr1*^flox/flox^ mice) [92]. The *Emx1-Cre;Itpr1*^flox/flox^ mice and *Gpr88-Cre;Itpr1*^flox/flox^ mice were born normally and showed normal growth patterns until adulthood, without apparent dyskinesia like total *Itpr1*^-/-^ mice. However, *Wnt1-Cre;Itpr1*^flox/flox^ mice began to show ataxia at around P9, and exhibited dyskinesia from 2 weeks after birth [92] (Table 2; Supplementary Table S1). *Wnt1-Cre;Itpr1*^flox/flox^ mice show abnormal cerebellar Purkinje cell (PC) firing patterns, due to altered PC activity [92]. *Itpr1* deficiency induces a series of abnormal electrophysiological features, including failed LTD in the PCs [241], and excessive LTP induction, attenuated depotentiation and LTP suppression, and altered presynaptic activity in hippocampal CA1 neurons [242,243]. However, typical ASD-related behaviors such as social and repetitive behaviors have not been investigated using these mice.

As enhanced PC activity and dystonia were observed in *Wnt1-Cre;Itpr1^flox/flox^* mice, pharmacological inactivation of cerebellar activity by AMPA receptor antagonist (CNQX) infusion was examined for therapeutic effects. Indeed, CNQX ameliorates the dyskinesia in these mice [92] (Table 3). Furthermore, dystonic movements were completely absent in *Wnt1-Cre;Itpr1*^flox/flox^ mice with genetic deletion of cerebellar PCs, achieved by mating *Wnt1-Cre;Itpr1^flox/flox^* mice with *Lurcher* mice (*GluD2*^LC/+^) to kill most of PCs by a mutation of the delta 2 glutamate receptor (GluD2) [92] (Table 3).

### 5.5. PRKCA (SFARI Gene Score: 3, Suggestive Evidence)

Protein kinase C alpha (PKC-α) is a member of lipid-sensitive serine/threonine protein kinases that regulate various cellular functions including proliferation, migration, adhesion, differentiation, and apoptosis. PKC-α appears to be a common downstream effector of RhoA, Cdc42, Rac1. *PRKCA* is located on chromosome 17q24.2. In two WES studies, three de novo missense variants in *PRKCA* were reported in ASD probands [95,161]. PKC-α has a variable regulatory domain at the N-terminus consisting of a C1 domain and a C2 domain, which function as the binding sites of diacylglycerol (DAG) and Ca^2+^, respectively. PKC-α also has a highly conserved kinase domain at the C-terminus comprised by a smaller ATP-binding loop and a substrate-binding site [244,245] (Figure 2C). PKC-α is ubiquitously expressed in all tissues, including the heart, adrenal gland, testis, lung, kidney, spleen, and liver, and is also widespread in various regions of the brain [246]. PKC-α is enriched in both neuronal and glial cultured cells [246]. A *PKC-α* KO mouse line has been generated, which appears to be normal with regard to external characteristics, viability, and fertility [247]. However, ASD-related behavioral analysis of these mice has not been reported.

### 5.6. WASF1 (SFARI Gene Score: S, Syndromic)

The Rac1 effector WAS protein family member 1 (WASF1), also known as WAVE-1/Scar1, is a member of the WASP-family. *WASF1* is located on chromosome 6q21. Using exome sequencing and whole-genome sequencing, three de novo truncated mutations of *WASF1* were reported in five unrelated individuals, all of whom presented ID with autistic features and seizures [248]. This is the only study so far that has reported the relationship between *WASF1* and ASD. WAVE-1 is composed by a Scar homology domain, a basic domain, a proline-rich region, a WASF homology (WH) domain, and an acidic domain [249] (Figure 2C). In human tissues, the expression of WAVE-1 is restricted to the brain [250]. Mouse study reveals that WAVE-1 shows high expression in the hippocampus, cortex, hypothalamus, amygdala, and cerebellum [93,251,252]. Two mouse lines of homozygous *Wave1* deletion are reported to be either postnatal lethal [251] or reduced in body size of offspring [93]. Behavioral analysis using *Wave1* KO mice (WAVE-1 null mice) generated by the second strategy showed hypoactivity, impaired motor coordination and balance, reduced anxiety levels, and defected spatial, nonspatial, and emotional learning and memory in the mice [93] (Table 2; Supplementary Table S1).

## 6. Conclusions

In this review, we summarize the findings of 20 ASD-risk genes of Rho GTPase regulators and effectors mainly from their mouse models. Most of these 20 genes are highly expressed in the hippocampus, cortex, amygdala and cerebellum in adult brains. These brain regions are important for behaviors such as social interaction and emotional regulation, and evidence from neuroimaging studies shows altered activation patterns in these regions in ASD individuals [253]. Therefore, mutations of Rho GTPase regulators and effectors may cause impaired neural function in these brain regions and thus abnormal behaviors in ASD individuals. Regarding cellular function, 13 of these genes (*Arhgef9*, *Trio*, *Prex1*, *Dock1*, *Dock4*, *Myo9b*, *Ophn1*, *Arhgef32*, *SrGAP3*, *Cyfip1*, *Pak2*, *Itpr1*, and *Wave1*) play important roles in regulating neuronal development and function, including axon guidance, dendrite and spine morphogenesis, and synaptic plasticity [60,67,68,71,80,89,92,135,140,150,224,251]. On the other hand, Dock8 and Myo9b are highly expressed in immune cells. In the nervous system, Dock8 regulates microglia migration [121] and Myo9b may have special function in microglia and astrocytes. It supports the view that microglia and astrocytes modulate synaptic function and contribute to the pathophysiologies of ASD [254,255].

In particular, this review summarizes the recent advances of behavioral and biological phenotypes observed using genetic mouse models of these genes. Most of these 20 genes have mouse models, and multiple behavioral paradigms have been tested in 13 mouse model lines. Notably, seven of these 13 mouse model lines (*Trio* cKO, *Prex1* KO, *Dock4* KO, *Arhgef10* KO, *Ophn1* KO, *Argap32* KO, and *Pak2* HET mice) exhibit impaired social behaviors and/or repetitive behaviors (Table 2; Supplementary Table S1), which are the core ASD symptoms. Some mouse models, such as *Arhgef9* KO mice and *Wave1* KO mice, were generated before the genes were found to be related to ASD, therefore ASD-related behavior analyses have not been done in these mouse models and will need to be investigated. Moreover, for some genes, such as *DOCK1*, *ARHGEF5*, and *NCKAP1*, homozygous KO mice are lethal due to severe developmental failure, so their roles in neuronal function and behaviors have not been explored in vivo. For some genes, such as *DOCK8* and *MYO9b*, genetic models have not been studied. Besides core symptoms of ASD, other closely related behavioral defects, presented in ASD-comorbid psychiatric disorders such as ID, ADHD, SCZ, are commonly observed in 13 mouse model lines. These include altered anxiety levels and poor learning and memory ability (Table 2; Supplementary Table S1). Findings from these mouse models contribute substantially to the understanding of Rho family GTPase-involved molecular pathogenesis of ASD.

Eight of the abovementioned 13 mouse model lines show synaptic transmission dysfunctions in hippocampal neurons, but the changes vary in different models. Some of the changes may be attributable to the common alteration of a particular Rho GTPase function. For instance, LTP is decreased upon deficiency of the Rac1 GEF Dock4 (*Dock4* KO mice) and the Rac1 effector Pak2 (*Pak2* HET mice), but is increased when the RhoA effector IP_3_R1 is deleted (*Itpr1* KO mice). Moreover, *Arhgef9* KO and *Prex1* KO mice, leading to inhibited Cdc42 and Rac1, respectively, fail to induce LTD. *Arhgef9* KO mice also show decreased GABAergic synaptic function, which is oppositely enhanced in the Rac1 effector *Cyfip1* cKO mice. These findings are consistent with the notion that RhoA and Rac1/Cdc42 may play opposite roles in modulating synaptic function. However, many factors, such as Ophn1 and Cyfip1, have additional functions besides participating Rho GTPase pathways. Altered activity of single Rho GTPase may not simply account for all the abnormalities in these mice. Nonetheless, these findings demonstrate that dysfunctions of Rho GTPase regulators and effectors lead to either too much or too little neurotransmission, supporting the hypothesis that impaired excitatory/inhibitory synaptic balance may be one of the pathogenic mechanisms generating ASD-like behaviors.

To investigate the possibility of treating the phenotypes in ASD mouse models, various strategies have been tried, which include genetic manipulation, cellular therapy, pharmacological intervention, and environmental stimulation [256]. We summarize the current successful treatments for mouse models of Rho GTPase regulators and effectors, including pharmacological and non-pharmacological approaches (Table 3). Interestingly, some strategies show similar therapeutic effects in different mouse models. For example, *Dock4* KO and *Prex1*^-/-^ mice both have reduced hippocampal Rac1 activity and show disruptive NMDA receptor function and social defects. Therefore, similar therapeutic strategies, including drug treatment to restore NMDA receptor function or replenishing Rac1 activity, rescue the defects in neural function and behaviors in both mice. In another case, both *Trio* cKO and *Ophn1* KO mice show increased PKA activity in the cortex, and the abnormal phenotypes due to *Trio* or *Ophn1* deficiency can be restored by the PKA inhibitor Rp-cAMPS treatment. Moreover, a dual inhibitor of PKA and ROCK, fasudil, also has therapeutic effects to treat the behavioral defects in *Ophn1*^-/y^ mice. Other strategies, such as GABA receptor agonist, AMPA receptor antagonist, and mGluR inhibitors, show treatment effects in different individual mice models. These lines of evidence suggest that personalized therapy, through either single or multiple therapeutic strategies, could be possible for patients who share common or unique pathologies due to their genetic variations.

## Figures and Tables

**Table 1:** Rho family GTPases involved in ASD.

ASD Candidate Gene	Gene Name	Chromosome Location	Genetic Category	SFARI Gene Score	Upstream/DOWNSTREAM Rho GTPase(s)
**Rho GTPase GEF**					
*ARHGEF9*	Cdc42 guanine nucleotide exchange factor (GEF) 9	Xq11.1-q11.2	Rare Single Gene Mutation, Syndromic	Category 1 (High Confidence)	CDC42
*TRIO*	Trio Rho guanine nucleotide exchange factor	5p15.2	Rare Single Gene Mutation, Syndromic	Category 1 (High Confidence)	RHOA, RAC1
*DOCK8*	Dedicator of cytokinesis 8	9p24.3	Rare Single Gene Mutation	Category 2 (Strong Candidate)	CDC42
*PREX1*	Phosphatidylinositol-3,4,5-trisphosphate-dependent Rac exchange factor 1	20q13.13	Genetic Association	Category 2 (Strong Candidate)	RAC1
*ARHGEF10*	Rho guanine nucleotide exchange factor 10	8p23.3	Rare Single Gene Mutation, Functional	Category 3 (Suggestive Evidence)	RHOA
*DOCK1*	Dedicator of cytokinesis 1	10q26.2	Rare Single Gene Mutation	Category 3 (Suggestive Evidence)	RAC1
*DOCK4*	Dedicator of cytokinesis 4	7q31.1	Rare Single Gene Mutation, Genetic Association, functional	Category 3 (Suggestive Evidence)	RAC1
**Rho GTPase GAP**					
*MYO9B*	Myosin IXB	19p13.11	Rare Single Gene Mutation	Category 2 (Strong Candidate)	RHOA
*OPHN1*	Oligophrenin 1	Xq12	Rare Single Gene Mutation, Syndromic	Category 2 (Strong Candidate)	RHOA, RAC1, CDC42
*ARHGAP5*	Rho GTPase activating protein 5	14q12	Rare Single Gene Mutation	Category 3 (Suggestive Evidence)	RHOA, RAC1, CDC42
*ARHGAP11B*	Rho GTPase activating protein 11B	15q13.2	Rare Single Gene Mutation	Category 3 (Suggestive Evidence)	Unknown
*ARHGAP32*	Rho GTPase activating protein 32	11q24.3	Rare Single Gene Mutation, Functional	Category 3 (Suggestive Evidence)	RHOA, RAC1, CDC42
*SRGAP3*	SLIT-ROBO Rho GTPase activating protein 3	3p25.3	Rare Single Gene Mutation	Category 3 (Suggestive Evidence)	CDC42, RAC1
*OCRL*	Oculocerebrorenal syndrome of Lowe	Xq26.1	Rare Single Gene Mutation, Syndromic	Syndromic	CDC42, RAC1
**Rho GTPase Effector**					
*NCKAP1*	NCK-associated protein 1	2q32.1	Rare Single Gene Mutation	Category 1 (High Confidence)	RAC1
*CYFIP1*	Cytoplasmic FMR1 interacting protein 1	15q11.2	Rare Single Gene Mutation, Genetic Association, Functional	Category 2 (Strong Candidate)	RAC1
*PAK2*	p21 (RAC1) activated kinase 2	3q29	Rare Single Gene Mutation	Category 2 (Strong Candidate)	CDC42, RAC1
*ITPR1*	Inositol 1,4,5-trisphosphate receptor type 1	3p26.1	Rare Single Gene Mutation	Category 3 (Suggestive Evidence)	RHOA
*PRKCA*	Protein kinase C alpha	17q24.2	Rare Single Gene Mutation	Category 3 (Suggestive Evidence)	RHOA, RAC1, CDC42
*WASF1*	WAS protein family member 1	6q21	Syndromic	Syndromic	RAC1

**Table 2:** Summary of ASD-related behavior tests in Rho guanine nucleotide exchange factor (GEF), GTPase-activating protein (GAP), and effector mouse models.

Gene	Mouse Model	Core Symptoms			Comorbidities				Reference
		Social Related Behavior	Language Communication	Repetitive Behavior	Anxiety and Depression	Learning and Memory	Basic locomotion and Motor Coordination	Schizophrenia and Epilepsy	
**Summary of ASD-related behavior tests in Rho GEF mouse models**									
*ARHGEF9*	*Arhgef9* KO mice *	NT ^1^	NT	NT	Anxiety ↑	Spatial learning and memory ↓	Activity ⎻	NT	[63]
*TRIO*	*Emx1-Trio*^−/−^ mice ^#^	NT	NT	NT	NT	Spatial learning and memory ↓ Fear memory ↓	NT	NT	[66]
	*NEX-Trio*^+/−^ mice ^&^	Social preference ↓	NT	Nestlet shredding (M^2^, ↑; F^3^, ⎻)	Anxiety ↑ Depression ⎻	Object recognition memory ⎻	Activity ↓ Motor coordination ↓	Prepulse inhibition ⎻	[67]
	*NEX-Trio*^−/−^ mice ^&^	Social preference ↓	NT	Nestlet shredding (M, ↑; F, ⎻)	Anxiety (M, ↑; F, ⎻) Depression ↑	Object recognition memory ⎻	Activity ↑ Motor coordination ↓	Prepulse inhibition (M, ↓; F, ⎻)	
*PREX1*	*Prex1*^−/−^ mice *	Social preference ↓ Social learning and memory ↓ Olfactory function ⎻	Ultrasonic vocalizations (pup) ↓	Grooming ↑	Anxiety ⎻	Reversal learning ↓ Fear memory ↓ Object recognition memory ⎻	Activity ⎻ Motor coordination ⎻	Prepulse inhibition ⎻	[68]
*ARHGEF10*	*Arhgef10* KO mice *	Sociability and social novelty preference ↓	NT	NT	Anxiety ↓ Depression ↓	Spatial learning and memory ⎻	Activity ↑	Prepulse inhibition ⎻	[69]
*DOCK4*	*Dock4* KO mice ^&^	Social novelty preference ↓	Ultrasonic vocalizations (pup) ↓	Stereotyped circling (~9% F; M, ⎻) Marble burying (M, ⎻; F, NT) Grooming (M, ⎻; F, NT)	Anxiety ↑	Object recognition memory (F, ↓; M, ⎻) Spatial memory (M, ↓; F, ⎻) Working memory (M, ↓; F, ⎻)	Activity (~9% F, ↑; M, ⎻)	NT	[70]
	*Dock4* HET mice ^&^	Social novelty preference (F,↓; M, ⎻)	Ultrasonic vocalizations (pup) ⎻	Stereotyped circling (~1.7% F; M, ⎻) Marble burying (M, ⎻; F, NT) Grooming (M, ⎻; F, NT)	Anxiety ⎻	Object recognition memory ⎻ Spatial memory (F, ↓; M, ⎻) Working memory ⎻	Activity (~1.7% F,↑; M, ⎻)	NT	
**Summary of ASD-related behavior tests in Rho GAP mouse models**									
*OPHN1*	*Ophn1*^-/y^ mice *	Aggressivity ↓ Social memory ⎻ Olfactory function ↓	NT	NT	Anxiety ⎻	Working, object recognition, and spatial learning and memory ↓ Fear memory extinction ↓ Vicarious trial and error (VTE) behavior ↓	Activity ↑ Motor coordination ⎻ Behavioral lateralization ↓	NT	[71,72,73,74,75]
*ARHGAP32*	*PX-RICS*^−/−^ mice (M were used in most behavior tests unless otherwise stated)	Social novelty preference ↓ social interaction ↓	Ultrasonic vocalizations (M and F,↓)	Grooming ↑ Marble burying ↑	NT	Reversal learning ↓ Fear memory ↓	Motor coordination ↓	Epilepsy (Severe progressive seizures)	[76,77]
	*PX-RICS*^+/−^ mice (M were used in most behavior tests unless otherwise stated)	Social novelty preference ↓ social interaction ↓	Ultrasonic vocalizations (M and F, ⎻)	Grooming ⎻ Marble burying ↑	NT	Reversal learning ⎻	Motor coordination ⎻	NT	[76]
*SRGAP3*	*WRP*^−/−^ mice ^&^	NT	NT	NT	Anxiety ⎻	Object recognition and long-term memory ↓ Spatial and reversal learning ↓ Working memory ⎻	Activity ⎻ Motor coordination ⎻	NT	[78]
	*WRP*^+/−^ mice ^&^	NT	NT	NT	Anxiety ⎻	Object recognition and long-term memory ↓ Spatial and reversal learning ↓ Working memory ⎻	Activity ⎻ Motor coordination ⎻	NT	
	*SrGAP3*^-/-^ mice ^&^	Social interaction ↓	NT	Marble burying (M, ⎻; F, NT)	Anxiety ⎻	Working memory ↓ Spatial and object recognition memory ⎻ Fear memory ↑	Activity (M,↓; F, ⎻)	Prepulse inhibition (F,↓; M, ⎻)	[79,80]
*OCRL*	*Ocrl1*^−/y^ mice *	NT	NT	NT	NT	Passive avoidance preference ⎻	Activity ⎻ Motor coordination ↓	NT	[81]
	*Ocrl1*^−/y^ mice * (*Inpp5b* deleted but human *INPP5B* overexpressed)	Social preference ⎻ Social novelty ⎻	NT	NT	NT	Spatial learning and memory ⎻	Activity ↓	NT	[82]
**Summary of ASD-related behavior tests in Rho effector mouse models**									
*CYFIP1*	*Cyfip1* HET mice *	Social interaction ⎻	NT	NT	Anxiety ⎻	Hippocampus-dependent memory ↓ Working, spatial, and fearing memory ⎻	Activity ⎻	Prepulse inhibition ⎻	[83]
	*Cyfip1*^HET^ mice ^#^	Social interest ↓	Ultrasonic vocalizations ⎻	Marble burying ⎻	NT	NT	Activity ⎻ Motor coordination ↓	NT	[84]
	*Cyfip1* m+/p− (Paternal origin) and *Cyfip1* m−/p+ (maternal origin) mice ^#^	NT	NT	NT	Anxiety-like behavior	Fear memory (m+/p−, ↑; m−/p+, ⎻)	Activity (m+/p−, ⎻; m−/p+, ↓)	NT	[85]
	*Cyfip*1^+/−^ mice *	NT	NT	Self-grooming ⎻ Marble burying ⎻	NT	Spatial memory and flexibility ⎻ Object recognition memory ↓ Working memory ⎻	Activity ⎻ Motor coordination ↓	Prepulse inhibition ↓	[86]
	*Cyfip*1^+/−^ rat *	NT	NT	NT	NT	Behavioral flexibility ↓	NT	NT	[87]
	Human *CYFIP1* overexpressing mice (Tg line 1 and Tg line 2) ^&^	Social preference ⎻	Ultrasonic vocalizations ⎻	Grooming ⎻ Digging ⎻	Anxiety ⎻	Fear memory (Line 1 and line 2, ↑) Spatial learning memory (Line 2, ↓; line 1, ⎻) Working memory (M and F of both lines, ⎻)	Activity ⎻	Prepulse inhibition ⎻	[88]
*PAK2*	*PAK2*^+/−^ mice *	Social preference ↓ Social memory ↓	Ultrasonic vocalizations ⎻	Marble burying ↑ Grooming ↑	Anxiety ⎻	Spatial learning and memory ⎻	Activity ⎻	Prepulse inhibition ⎻ Acoustic startle response ⎻	[89]
*ITPR1*	*IP3R1*^+/−^ mice^&^	NT	NT	NT	NT	NT	Activity ⎻ Motor coordination ↓	NT	[90]
	*L7-Cre*; *Itpr1*^flox/flox^ mice ^#^	NT	NT	NT	NT	NT	Motor coordination ↓	NT	[91]
	*Wnt1-Cre*; *Itpr1*^flox/flox^ mice ^#^	NT	NT	NT	NT	NT	Motor coordination ↓	NT	[92]
*WASF1*	*WAVE-1* KO mice ^#^	NT	NT	NT	Anxiety ↓	Spatial learning and memory ↓ Object recognition memory ↓ Passive avoidance ⎻	Activity ↓ Motor coordination ↓	NT	[93]
	*WAVE-1* HET mice ^#^	NT	NT	NT	Anxiety ⎻	Learning and memory ⎻	Activity ↓ Motor coordination ↓	NT	

*: Only male were used in behavior tests; ^&^: Both male and female mice were used in behavior tests; ^#^: Mice gender was not mentioned; ^1^ NT: not tested; ^2^ M: male mice; ^3^ F: female mice;↑ is increased and ↓ is decreased; − is no change; More detailed information is shown in Supplementary Table S1.

**Table 3:** Treatments for Rho GTPase mouse models.

Gene	Mouse/Cellular Model	Therapeutic Type	Therapeutic Strategy	Result	Reference
*TRIO*	*Trio* deficient neurons	Pharmacological	Rp-cAMPS treatment (100 μM)	Increased dendritic spine density reversed	[67]
		Non-pharmacological	PDE4A5 overexpression		
*P-REX1*	*Prex1*^-/-^ mice	Pharmacological	D-serine (for electrophysiology: 20 μM; for mouse: 0.8 g/kg i.p.(intraperitoneal))	NMDAR-LTD restored; disruptive social novelty corrected	[68]
		Non-pharmacological	WT P-Rex1 or WT Rac1 overexpression (in CA1 pyramidal neurons)	NMDAR-LTD restored; disruptive social novelty and reversal learning corrected	
*DOCK4*	*Dock4* KO mice	Pharmacological	D-cycloserine (DCS, 20 mg/kg i.p.)	Social novelty restored	[70]
		Non-pharmacological	WT Rac1 overexpression (in CA1 region)	Social novelty and synapatic transmission (mEPSC and LTP) restored	
	*Dock4* knockdown neurons	Non-pharmacological	WT Rac1 overexpression	Decreased dendritic spine density reversed	[109]
*OPHN1*	*Ophn1*^-/y^ mice	Pharmacological	Rp-cAMPS (bilaterally infused into PFC; 10 μg/μL; 300–400 nl)	Cognitive dysfunction in Y-maze ameliorated	[75]
			Fasudil (dissolved in daily drinking water at 0.65 mg/mL for 3 weeks)	Spine morphology in olfactory bulbs, frequency and amplitude of mIPSC in olfactory neurons, and olfactory behaviors rescued	[73]
				Fear memory extinction restored	[74]
			Fasudil (orally a daily dose of 3 mg for 3 months)	Locomotor activity and object recognition memory restored; abnormal brain morphology ameliorated	[72]
*ARHGAP32*	*PX-RICS*^-/-^ mice	Pharmacological	Clonazepam (CZP, 0.03 mg/kg i.p.)	Deficits of social preference, reversal learning, and cued fear learning memory reversed	[76,77]
*CYFIP1*	*Cyfip1* HET mice hippocampal slices	Pharmacological	LY367385 (100 μM) and MPEP (2-Methyl-6-phenylethynyl pyridine), (10 μM) (Incubated in slices)	mGluR-LTD normalized to control levels	[83]
	*Cyfip1*^HET^ mice	Non-pharmacological	Motor training (at postnatal days 40, 50, and 51)	Motor deficits alleviated	[84]
*PAK2*	*Pak2*^+/-^ mice	Non-pharmacological	p-cofilin peptide (15 pmol/g i.v. (intravenous))	Social behaviors moderately improved	[89]
*ITPR1*	*Wnt1-Cre;Itpr1*^flox/flox^ mice	Pharmacological	CNQX (5 mM; infused into the cerebellum; 0.5 μL/min for 20 min)	Dyskinesia ameliorated	[92]
		Non-pharmacological	Mating with *Lurcher* mice (*GluD2*^LC/+^)	Dystonic movements eliminated	

## Supplementary Materials

The following are available online at https://www.mdpi.com/2073-4409/9/4/835/s1, Table S1: Summary of ASD-related behavior tests in Rho GEF, GAP, and effector mouse models.

## Appendix A

The order of genes discussed in the review is according to SFARI Gene scores which reflect the gene linkage strengths to ASD; Genes of same score are listed alphabetically.
